# Recurrent, non-traumatic, non-exertional rhabdomyolysis after immunologic stimuli in a healthy adolescent female: a case report

**DOI:** 10.1186/s12887-022-03561-2

**Published:** 2022-08-30

**Authors:** Jason Katz, Anatalia Labilloy, Andrew Lee

**Affiliations:** 1 College of Medicine, UF College of Medicine, Gainesville, FL 32610 USA; 2 Department of Pediatrics, Division of Pediatric Genetics, UF College of Medicine, Jacksonville, FL 32207 USA; 3 Department of Pediatrics, Division of General Pediatrics, UF College of Medicine, Jacksonville, FL 32217 USA

**Keywords:** Rhabdomyolysis, COVID-19 mRNA vaccine, Dysferlinopathy, Human papilloma virus vaccine, Group A Streptococcal infection, Whole exome sequencing, Limb-girdle muscular dystrophy type 2B, Miyoshi myopathy, Asymptomatic hyperCKemia, Distal myopathy with anterior tibial onset, Case report

## Abstract

**Background:**

Dysferlinopathy refers to a heterogenous group of autosomal recessive disorders that affect a skeletal muscle protein called dysferlin. These mutations are associated with limb-girdle muscular dystrophy type 2B, Miyoshi myopathy, asymptomatic hyperCKemia, and distal myopathy with anterior tibial onset.

**Case presentation:**

A 16 year old female presented with myalgia, weakness and dark urine one week after her second BNT162b2 mRNA (Pfizer) vaccine. Initial serum creatine kinase (CK) was measured at 153,000 IU/L, eventually up-trending to over 200,000 IU/L. However, stable renal function precluded hemodialysis allowing discharge after 10 days of intravenous (IV) hydration and alkaline diuresis.

Just two years prior to the current presentation, the patient was hospitalized following Group A Streptococcal pharyngitis infection complicated by rhabdomyolysis. She presented with fatigue, lower extremity weakness, and dark oliguria with CK measuring 984,800 IU/L. IV hydration was attempted however hemodialysis was ultimately required throughout her 24-day hospital stay. Her episode was presumed to be idiopathic and no further work-up was performed at that time.

During the patient’s current hospitalization, she reported similar symptomology (myalgias and weakness) following her first quadrivalent Gardasil vaccine at age 11. No hospitalization was required at that time. A comprehensive workup was now initiated while the patient was being treated for her suspected second or third non-exertional, non-traumatic rhabdomyolysis. Rheumatologic, metabolic, infectious, and endocrinologic workup were all unremarkable. Patient eventually had whole exome sequencing performed which revealed a heterozygous pathogenic variant in the *DYSF* gene (*DYSF* c.2643 + 1G > A) encoding dysferlin. No clinically significant sequelae occurred thus far.

**Conclusions:**

While there have been reports of symptomatic heterozygote carriers of dysferlinopathies, to our knowledge none have been associated with recurrent rhabdomyolysis after immunogenic stimuli. This unique case presentation highlights the importance of a multi-disciplinary care team, the utility of modern whole-exome gene sequencing, and the future challenges of balancing vaccine risk vs benefit.

**Supplementary Information:**

The online version contains supplementary material available at 10.1186/s12887-022-03561-2.

## Background

Dysferlinopathy refers to a heterogenous group of autosomal recessive disorders that are caused by mutations in *DYSF* gene that encodes a skeletal muscle protein, dysferlin. With more than 300 sequence variations including both deleterious and nonpathogenic polymorphisms [1], diagnosis usually involves a detailed history and physical to elicit a clinical phenotype, along with muscle biopsy, Western blot, and imaging. Conditions with distinct phenotypic presentations that have been associated with DYSF gene mutations include limb-girdle muscular dystrophy type 2B (LGMD2B), Miyoshi myopathy (MM), asymptomatic hyperCKemia, and distal myopathy with anterior tibial onset (DMAT) [1]. While there have been reports of symptomatic heterozygote carriers of dysferlinopathies, to our knowledge none have been associated with recurrent rhabdomyolysis after immunogenic stimuli.

## Case presentation

### Initial presentation

A 16 year old African American female with a past medical history of obesity, depression, irregular menses, presumed idiopathic rhabdomyolysis, currently taken no medications presented to the emergency department (ED) with one week history of myalgia and muscle weakness in bilateral upper arms, thighs, and buttocks. Patient also endorsed dark urine starting the morning of presentation. This was reminiscent of a prior rhabdomyolysis hospitalization prompting the patient and her mother to immediately go to the ED.

**Fig. 1:**
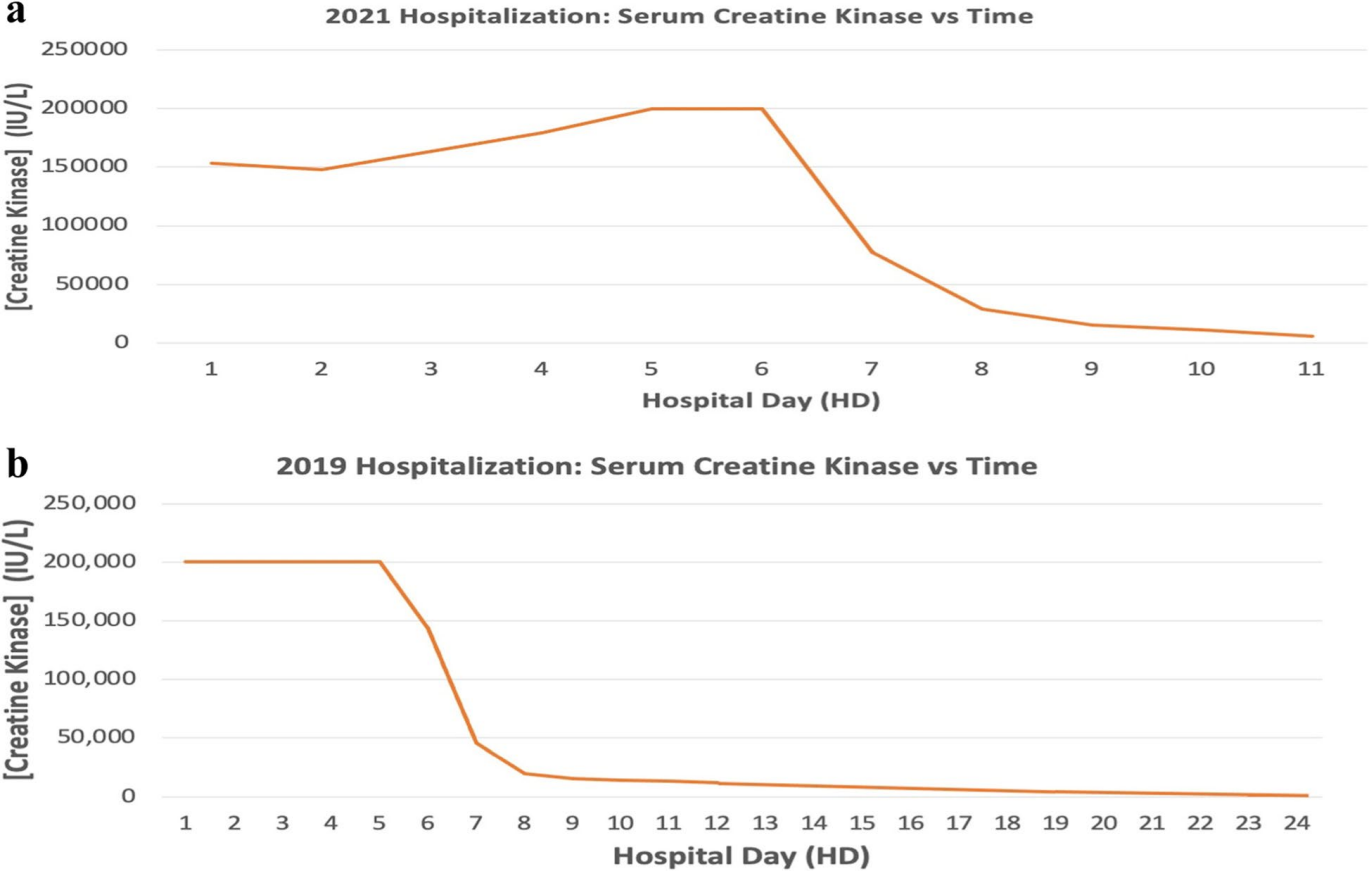
**a**-**b** Serum creatine kinase (CK) levels versus time during both rhabdomyolysis hospitalizations. Standard reported upper limit of detection for our laboratory testing was 200,000 IU/L thus samples were further diluted to achieve accurate value. These values are not reported here, however in both hospitalizations there were instances of CK well above 200,000 IU/L

Patient arrived hemodynamically stable with physical exam rather unremarkable aside from diffuse myalgias and muscle weakness. The patient exhibited normal range of motion with pain with passive movement of the bilateral upper and lower extremities (UE/LE). Initially she had 4/5 strength in bilateral UE and LE with improvement to 5/5 strength in bilateral UE and LE by hospital discharge. Cranial nerves II-XII were grossly intact with bilateral knee deep tendon reflexes 2/4. Her cerebellar tests were unremarkable. Serum creatine kinase (CK) was measured at 153,000 IU/L (Fig. 1a) with urinalysis showing large “blood” and protein (100 m/dL). No other serum electrolyte abnormality was noted. Ionized calcium was 4.4 mg/dL with serum calcium 7.2 mg/dL and albumin 2.7 g/dL. Electrocardiogram showed sinus tachycardia with QTc of 443 ms.

### History

Patient reported that she received her second dose of BNT162b2 mRNA COVID-19 vaccine the week prior to presentation. There were reported expected side effects at time of injection (i.e. pain at injection site, headache, and fatigue). Several days later, she began experiencing myalgias in her bilateral arms, thighs, and buttocks unresponsive to acetaminophen and ibuprofen. Patient denied any fever, chest pain, shortness of breath, nausea, vomiting, diarrhea, abdominal pain, rash, sore throat, dysuria, dizziness, headache. Patient also denied any recent strenuous activity including marathon, weight lifting, hiking etc. Reported family history was significant for thyroid disease and glaucoma in her biological mother as well as hypertension and congestive heart failure (CHF) in her biologic father.

Two years prior, the patient was hospitalized following febrile Group A Streptococcal (GAS) pharyngitis infection complicated by rhabdomyolysis. At that time, she presented with fatigue, lower extremity weakness, and dark urine. She was being treated with amoxillin-clavulanic acid (500-125 mg) and prednisone (10 mg) following a 1 week history of sore throat and fever. The patient’s inability to ambulate and diminished urinary output prompted her arrival to the ED. Vital signs were unremarkable except for “mild erythematous tonsils without exudate” and tenderness over “bilateral anterior thighs”. Initial laboratory studies showed a CK of 984,800 IU/L (Fig. 1b), potassium of 7.0 mEq/L, blood urea nitrogen of 31 mg/dL, creatinine of 2.26 mg/dL, aspartate aminotransferase (AST) of 2550 IU/L, alanine aminotransferase (ALT) 601 Iu/L, calcium 4.9 mg/dL, white blood cell 15,700 K/mcL with polymorphonuclear cells 87%, 7% lymphocytes. Electrocardiogram (ECG) showed peaked T waves. Patient was immediately transferred to pediatric intensive care unit (PICU) due to aberrant physiological parameters.

The patient’s hospital course included aggressive intravenous (IV) fluid resuscitation, IV penicillin, correction of electrolyte and metabolic acidosis derangements, and forced alkaline diuresis with sodium bicarbonate. Despite these measures, patient required multiple rounds of hemodialysis throughout her 24 day hospital stay along with intense physical therapy. There were no other major complications aside from simple cystitis urinary tract infection (UTI) treated with combination of IV/oral antibiotics. The patient was successfully discharged with normal range CK (Fig. 1b) after being able to void greater than 1.5 L of urine for continuous 5 days before discharge.

Upon further history taking, patient reported that after her first quadrivalent human papillomavirus (HPV) vaccine (Gardasil, 4vHPV) in 2014, she experienced similar symptomology: severe diffuse myalgia and muscle weakness. However, this incident did not require hospitalization and patient was unable to remember if her urine was dark.

### Current rhabdomyolysis hospitalization after 2^nd^ BNT162b2 mRNA SARS-CoV-2 vaccine

The patient’s current rhabdomyolysis was treated in a similar fashion except no dialysis was needed during this hospitalization stay. Contemporaneously, an extensive work-up was initiated for possible etiology of this patient’s recurrent non-traumatic, non-exertional.

rhabdomyolysis. A multi-disciplinary team was consulted including nephrology, genetics, neuromuscular neurologists, and rheumatology. Per genetics recommendation, whole exome sequencing was ordered along with serum profiles of amino acids, urine organic acids, pyruvate levels, acylcarnitine, and carnitines. No electromyography (EMG), extended myositis panel, muscle biopsy or musculoskeletal MRI was performed while inpatient due to active rhabdomyolysis with low suspicion that results would change management.

Urine myoglobin was calculated at 12,700 mcg/L 15 h after ED presentation. CK up trended to over 200,000 IU/L by hospital day 3 (Fig. 1a), remaining there until hospital day (HD) 5. Throughout the hospital stay, patient had intermittent chest pain/ “muscle spasms” and tachypnea that the patient attributed to her anxiety. Cardiac evaluation with electrocardiogram, chest X-ray and transthoracic echocardiography was unremarkable.

Our patient was ultimately discharged after 10 days and CK = 5,848 IU/L (Fig. 1a) in stable condition on amlodipine for hypertension with normal renal function. No etiology was present at that time to explain her recurrent rhabdomyolysis as the patient’s serum profiles of amino acids, urine organic acids, pyruvate levels, acylcarnitine, and carnitines were largely unremarkable with whole exome genome sequencing still pending. The patient overall adhered and tolerated inpatient treatment well, however did require inpatient psychotherapy for mood symptoms.

### Post-hospitalization follow-up

One month after discharge, the patient was seen by outpatient rheumatology where an autoimmune work up was unremarkable (+ anti-nuclear antibody (ANA) however low titer at 1:140) with a physical exam “reassuring for no other signs of possible rheumatic disease”. Two months after discharge, the patient was seen by nephrology with no interval changes per patient, normal physical exam, and small amount of blood and trace leukocytes on point of care urinalysis.

Three months after discharge, patient was seen by genetics where exome sequencing resulted with a heterozygous pathogenic variant in the *DYSF* gene (*DYSF* c.2643 + 1G > A). The *DYSF*gene, found on chromosome 2p31, encodes a 230 kDa skeletal muscle sarcomere protein called dysferlin [2]. DYSF mutations have been associated with several autosomal recessive inherited conditions: limb-girdle muscular dystrophy type 2B (LGMD2B), Miyoshi myopathy (MM), asymptomatic hyperCKemia, and distal myopathy with anterior tibial onset (DMAT) [1].

Due to the presence of only one known pathologic allele variant, it does not yet establish a molecular diagnosis. One month after the genetics visit, the patient was seen by neurology where physical exam was unremarkable and CK and liver function tests were within normal limits. The patient and family was also advised to avoid strenuous physical activity while obtaining both a cardiology consult and targeted variant testing for first degree relatives [3].

## Discussion and conclusions

Dysferlinopathy refers to a heterogenous group of autosomal recessive disorders that affect DYSF gene, ultimately impacting a skeletal muscle protein, dysferlin. With more than 300 sequence variations including both deleterious and nonpathogenic polymorphisms [1], diagnosis involves a history and physical to elicit a clinical phenotype, muscle biopsy, Western blot, and imaging. Of the four previously mentioned autosomal recessive conditions associated with DYSF gene mutations (LGMD2B, MM, DMAT, and asymptomatic hyperCKemia), LGMD2B fits our patient’s illness script most closely due to proximal muscle predominance, teenage onset, massive CK elevation, and slow progression [3].

There remains diagnostic ambiguity in our case for several reasons: lack of family history, lack of myopathy and prominent muscle weakness/atrophy at baseline, and identification of only a single pathogenic variant. However, a lack of family history has shown to not preclude diagnosis [3] and there have been reports of symptomatic heterozygotes [4]. Furthermore, this patient may possess an additional variant on the other allele in a location undetectable by the sequencing technology. Nevertheless, it’s intriguing that each of her symptomatic episodes revolved around immunologic stimuli/stressors (HPV vaccine, GAS pharyngitis, COVID-19 vaccine) while prolonged exertion (i.e. 5 km race in elementary school) resulted in no issues.

### Other differentials for recurrent, non-traumatic, non-exertional rhabdomyolysis

Evidence suggests that rhabdomyolysis can have genetic underpinnings with environmental interactions [5, 6]. One example of this is the autoimmune/inflammatory syndrome induced by adjuvants (ASIA). Also known as Shoenfeld’s syndrome, it includes various autoimmune illness scripts (myalgia, arthralgias, fatigue, and neurological perturbations) surrounding an exposure to an adjuvant. These agents include oils, mineral salts, lipopolysaccharides, and peptidoglycan found in vaccines to trigger innate and adaptive responses [7]. Interestingly, quadrivalent human papillomavirus (HPV) vaccine (Gardasil, 4vHPV) contains amorphous aluminum hydroxyphosphate sulfate adjuvant and vaccination is associated with higher rates of arthritis, systemic lupus erythematosus, vasculitis, and central nervous system (CNS) conditions [8]. Macrophagic myofasciitis syndrome (MMF) [9] is under the ASIA syndrome spectrum and involves aluminum accumulation in macrophages between muscle fibers leading to autoimmune inflammation and myalgia, fatigue, and CNS symptoms (decreased memory, mood disturbances, attention deficit). Our patient did experience severe anxiety and depression both during and before her latest hospitalization however it is unclear if this is related to these syndromes or coincidental. Furthermore, the Pfizer-BioNTech mRNA BNT162b2 vaccine does not contain aluminum adjuvants and our patient did not have significant arthralgia or fatigue concerns. Nevertheless further research may elucidate if this novel mRNA and lipid nanoparticle design may have similar immunologic consequences in a genetically susceptible individual.

Metabolic causes of her rhabdomyolysis (i.e. inherited disorders of glycogenolysis, glycolysis, or lipid metabolism) were ruled out via normal plasma amino acids, urine organic acids, pyruvate levels, acylcarnitine, and total and free carnitines [10]. Other major etiologies of non-traumatic, non-exertional rhabdomyolysis that were ultimately ruled out include drugs, toxins, infections, electrolyte imbalances, endocrine disorders, seizures, and inflammatory myopathies (Table 1). Inflammatory myopathies including dermatomyositis and polymyositis were ruled out via negative antibody panels. Other rheumatologic work up was negative (Appendix).

**Table 1:** A helpful mnemonic our authors constructed to highlight various etiologies of rhabdomyolysis [11]

**R:** Rare genetic disorders	disorders of glycolysis, glycogenolysis, lipid metabolism, mitochondrial
**H:** Hormones	endocrine pathology including diabetes, hypo/hyperthyroidism, pheochromocytoma
**A:** Autoimmune inflammatory myopathies	dermatomyositis, polymyositis
**B:** Body temperature extremes	hyperthermia and hypothermia
**D:** Drugs	statins, colchicine, daptomycin, anesthetics, cocaine, alcohol, heroin, amphetamines, methadone, Lysergic acid diethylamide (LSD)
**O:** Other	ASIA (autoimmune/inflammatory syndrome induced by adjuvants), seizures, infections, trauma, exertion, ischemia

### Common trigger: immunologic stimuli?

Non-traumatic, non-exertional rhabdomyolysis in previously healthy young patients has been reported following BNT162b2 mRNA vaccination [12]. Severe debilitating reactive myalgia after GAS pharyngitis has also been documented [13], however serum creatine kinase was within normal range. Nevertheless, there is extensive literature that shows rhabdomyolysis can occur after infections including Group C beta-hemolytic streptococcal pharyngitis infection, Clostridium perfringens, pseudomonas, influenza, HIV, legionella, salmonella [14, 15]. While our group is unaware of reported associations between rhabdomyolysis and HPV vaccine, there is evidence that a heterogenous clinical syndrome is temporally associated with vaccination including fatigue, widespread pain, cognitive dysfunction, orthostatic intolerance, and chronic regional pain syndrome [7, 16]. Because the patient was not hospitalized following her myalgias after HPV vaccination, it is difficult to gather an objective assessment of her diagnosis and association with HPV vaccination.

It is worth mentioning that our discussion here should not be widely construed as an argument against COVID-19 and/or HPV vaccination. Multiple studies have shown the safety, efficacy, and limited serious side effect profiles of BNT162b2 mRNA vaccinations [17–20]. Additionally, HPV vaccination has shown to have tremendous harm reduction for cervical dysplasia and cancer [21] as well as head and neck squamous cell cancer [22]. Rather, our case report offers an interesting clinical observation of severe, recurrent rhabdomyolysis after various immunologic stimuli, most recently of which was after SARS-CoV2 vaccination.

### Future

In addition to genomic sequencing, muscle biopsy and subsequent western blot for dysferlin protein analysis is essential to characterize aberrant expression [1]. Muscle biopsy may show dystrophic changes and irregular quantity or distribution of dsyferlin on the sarcolemma [23]. This is complicated since normal dysferlin expression on Western blot analysis can occur even in a patient presenting with physical phenotype and genomic evidence of homozygous dysferlinopathy (Miyoshi myopathy) [1]. Human leukocyte antigen (HLA) typing may provide haplotypes (HLA DRB1, DQB1) known to be associated with ASIA-related conditions [7]. Muscle MRI may be an alternative, less-invasive method to characterize muscle abnormalities, as it has been used in symptomatic heterozygote carriers of limb girdle muscular dystrophy 2B and Miyoshi myopathy [23]. Additionally, subclinical involvement of other muscle groups (i.e. distal musculature) may be elucidated with muscle computed tomography (CT) and/or MRI which can aid in diagnostic accuracy and prognostication [3].

This unique case-presentation is important as it highlights the importance of a multi-disciplinary care team in forming a broad differential for recurrent rhabdomyolysis in an otherwise healthy, asymptomatic female adolescent. It shows the utility of modern clinical advancements in whole exome gene sequencing. It also highlights the future challenges that will undoubtedly be faced by patient, family and clinicians moving forward as they balance the potential risks and benefits of future vaccinations as well as unanswered questions regarding prognosis. 

## Supplementary Information

**Additional file 1:** **Appendix. **Comprehensive laboratory work-up.

## Data Availability

The datasets generated and/or analyzed during the current case report are not publicly available due to patient privacy but are available from the corresponding author on reasonable request.

## Abbreviations

IV: Intravenous
LGMD2B: Limb-girdle muscular dystrophy type 2B
MM: Miyoshi myopathy
DMAT: Distal myopathy with anterior tibial onset
CK: Creatine kinase
ASIA: Autoimmune/inflammatory syndrome induced by adjuvants
ED: Emergency department
GAS: Group A Streptococcal
CHF: Congestive heart failure
EMR: Electronic medical record
AST: Aspartate aminotransferase
ALT: Alanine aminotransferase
ECG: Electrocardiogram
PICU: Pediatric intensive care unit
UTI: Urinary tract infection
HD: Hospital day
EMG: Electromyography
MRI: Magnetic resonance imaging
HPV: Human papilloma virus
ANA: Anti-nuclear antibody
MMF: Macrophagic myofasciitis syndrome
GWS: Gulf War Syndrome
CNS: Central nervous system
LSD: D-lysergic acid diethylamide
TSH: Thyroid stimulating hormone
T4: Thyroxine
Ig: Immunoglobin
Ab: Antibody
SCL70: Anti-topoisomerase 1
dsDNA: Double-stranded DNA
U1-RNP: U1 small nuclear ribonucleoprotein
SS-A/Ro: Sjogren syndrome A
SS-B/La: Sjogren syndrome B
Jo1: Anti-histidyl tRNA synthetase
HLA: Human leukocyte antigen
CT: Computed tomography
