# Supplementary material for: Recurrent, non-traumatic, non-exertional rhabdomyolysis after immunologic stimuli in a healthy adolescent female: a case report
Source: BMC Pediatr. 2022 Aug 30;22:515. doi: 10.1186/s12887-022-03561-2 (PMC9426381; doi:10.1186/s12887-022-03561-2)
Supplement: Supplementary file 1 — Additional file 1: Appendix. Comprehensive laboratory work-up. [file 12887_2022_3561_MOESM1_ESM.docx]

**Appendix: Comprehensive laboratory work-up**

Rheumatologic work up: Inflammatory myopathies including dermatomyositis and polymyositis were ruled out along with other rheumatologic conditions via negative antibodies for anti-Smith immunoglobin (Ig) G antibody (Ab), anti-topoisomerase 1 (SCL70) Ab IgG, anti double-stranded DNA (dsDNA) Ab, anti U1 small nuclear ribonucleoprotein (U1-RNP) Ab, Sjogren syndrome A (SS-A/Ro) Ab, Sjogren syndrome B (SS-B/La) Ab, anti-histidyl tRNA synthetase (Jo1) Ab, and anti-centromere Ab. Though she did have a positive ANA, it was of low titer (1:140) thus not clinically significant. She also had no stigmata of rheumatologic conditions after history and physical examination by a rheumatologist.
